# Preference for Institutional Delivery and Caesarean Sections in Bangladesh

**DOI:** 10.3329/jhpn.v31i1.14754

**Published:** 2013-03

**Authors:** S.M. Mostafa Kamal

**Affiliations:** Department of Mathematics, Islamic University, Kushtia 7003, Bangladesh

**Keywords:** Caesarean sections, Delivery settings, Logistic regression, Pregnancy, Bangladesh

## Abstract

In Bangladesh, preference for place of delivery and socioeconomic factors associated with caesarean section are not well-understood. This paper examines the socioeconomic correlates of preference for institutional delivery and caesarean sections in Bangladesh. The study used data from the nationally-representative 2007 Bangladesh Demographic and Health Survey. Both bivariate and multivariate binary logistic regression models were constructed to assess the effect of sociodemographic factors on the use of medical facilities and caesarean section for childbirth. Overall, 15% of women underwent institutional delivery, and 8% deliveries were performed by caesarean sections. Both institutional deliveries and caesarean sections have increased in recent years. The bivariate and multivariate analyses both confirmed that place of residence, religion, birth order, frequent pregnancy, antenatal care-seeking, and wealth index were important predictors of the use of medical facilities and caesarean sections for childbirth. Women's education appeared as the most single significant determinant for the use of both services. The findings underlie the importance of monitoring caesarean section as well as professional attendance for safe motherhood. Programmes should aim to inform women highlighting the benefits of the use of skilled maternal healthcare services and demerits of home-delivery practices.

## INTRODUCTION

An estimated 358,000 maternal deaths occurred worldwide in 2008—a 34% decline from the level of 1990. Despite this decline, developing countries continue to account for 99% of the total maternal deaths. Of these estimated deaths, sub-Saharan Africa and South Asia accounted for 87% of the global maternal deaths. Overall, maternal mortality ratio (MMR) was the highest in developing regions (290/100,000) in stark contrast to developed regions (14/100,000). Bangladesh was one among the eleven developing countries, which had 65% of all maternal deaths. This implies that Bangladesh still has high MMR (340/100,000), which is one of the highest in the world (1).

Improving maternal health was one of the prior issues of the 2000 Millennium summit. Since the initiation of safe motherhood programme in 2001 with the signature of 189 countries in the Millennium declaration, in which Millennium Development Goal 5 (MDG 5) calls for a reduction in MMR by 75% and achieving universal access to reproductive health by 2015, the progress toward achieving the target of MMR has been uneven all over the world. For instance, among the developing regions, sub-Saharan Africa has the highest MMR, followed by South Asia, South Eastern Asia, North Africa, Latin America and the Caribbean, Western Asia, and Eastern Asia (1).

Globally, there is now consensus that increasing rates of delivery with a skilled attendant, ideally in a well-equipped facility, are essential to reduce maternal mortality (2,3). Appropriate delivery care is crucial for both maternal and perinatal health, and increasing skilled attendance at birth is a central goal of the safe motherhood and child survival movements (4). In addition, it is important that mothers should deliver in an appropriate setting where lifesaving equipment and hygienic conditions are available and can help reduce the risk of complications that may cause death or illness to the mother and the child (5).

Bangladesh has a long tradition of home-delivery practice. Delivery-related complication is one of the leading causes of maternal mortality in Bangladesh. Findings of a study conducted in rural Bangladesh showed that one-third of the women experienced delivery-related complications during their last delivery (6). The estimated lifetime risk of dying from pregnancy and childbirth-related causes in Bangladesh is about 100 times higher than that in the developed countries. The tragic consequence of these deaths is that about 75% of the babies born to these women die within the first week of their lives (7).

Surgical interventions during pregnancy are usually made to ensure safety of the mother and the child under conditions of obstetric risk. Although rates of caesarean section in many countries have increased from the recommended level of 15% in developed and many developing countries (3), the rate of delivery through caesarean section is relatively low in Bangladesh. The country still has very lower use-rate of the maternal healthcare services. Research based on 42 demographic and health surveys in developing countries evidently showed that, in the poorest countries, large proportions of the population have no access to potentially lifesaving caesarean section. Generally, financial costs play an important role in the demand for healthcare and for maternity care in particular (8,9,10). Apart from the clinical indications for caesarean section—breech presentation, dystocia, and suspected foetal compromise—there is growing evidence that many women choose delivery by caesarean section for personal reasons, particularly in profit-motivated institutional settings that may provide implicit or explicit encouragement for such interventions (11).

Caesarean section is a surgical procedure for delivery when vaginal delivery becomes contraindicated (12). The caesarean section is of benefit to pregnant women and the newborns when its indication is well-founded (13). Apart from medical indications, the reasons for the increase in the rates of caesarean delivery are stated as fear for vaginal delivery, multiple pregnancy, and the increase in the use of electronic foetal monitoring (12,14,15). In addition, the belief that caesarean delivery is safer for both mother and the baby turns women into the preferred childbirth through caesarean delivery (16).

Currently, there is much debate as to whether this surgical procedure should be performed for women without clear clinically-acceptable indications (17,18). Some studies recommend for vaginal delivery (19,20) whereas some others recommend for caesarean section (21). Studies reported that postpartum haemorrhage occurs most commonly in the vaginal delivery (20) whereas caesarean deliveries are associated with a higher rate of postpartum morbidity (19). In addition, compared to spontaneous vaginal delivery, caesarean delivery is associated with increased risk of endometritis, pneumonia, and the need for blood transfusion (20). It is also reported that the pelvic dysfunction, particularly peripartum risk factor, results from a combination of structural damage and neurologic injury that occurs during labour and, most certainly, during vaginal birth (22,23).

Bangladesh has a fairly extensive network of providing maternal and child health services from grassroots to higher levels (24). The female field workers make selective visits to pregnant women at the household level and motivate them to visit paramedics for antenatal check-ups at the community clinic (CC) or at the union-level Health and Family Welfare Centre (H&FWC). Each CC is meant for providing services to an average of 6,000 population, and an H&FWC covers, on an average, a population of 25,000. The upazila-level hospital, named Upazila Health Complex (UHC) (1 hospital for about 250,000 to 300,000 population), provides basic emergency obstetric care (EmOc) services, and refers clients with complications to the district-level hospital where comprehensive EmOc services are provided. However, despite the availability of EmOc services, the use of these services is still poor (24).

Delays are often experienced in recognizing a case of emergency for deciding to seek treatment and travelling to treatment facilities. Two in five women cannot decide whether to seek treatment within six hours of recognizing complications. For one-fifth of those who decided to go to the health facility, the travel time was more than one hour (25). There are often delays in actually receiving treatment at the facility, and the cost of treatment is another deterrent for many people in Bangladesh where 49.6% of the population lives on less than US$ 1.25 a day, and 40% people live below the poverty line (26). Inadequate obstetric and neonatal care provided by health facilities also adds to the problem. Poor quality of services precludes optimal utilization of services. Lack of skilled healthcare providers is a major problem both at facility and community levels. Referral services are not always accessible or simply available. Provision of health services is unequal across the regions, and coordination between child and maternal health programmes is often absent. Reduction in the number of maternal deaths requires timely access to effective, affordable and appropriate EmOc services (27).

So far known, a few studies have been conducted on women's preference for delivery settings and factors associated with delivery through caesarean section in Bangladesh. This study aims to explore the socioeconomic correlates of modes of delivery and caesarean section performed in the last five years preceding the survey, using dataset of a nationally-representative survey. Since there is lack of studies on this area, the information on socioeconomic inequality in service-use for facility-based delivery and caesarean section is potentially significant.

## MATERIALS AND METHODS

### Data

The study used data from 2007 Bangladesh Demographic and Health Survey (BDHS). The 2007 BDHS is a nationally-representative survey of 10,996 women aged 15-49 years and 3,771 men aged 15-54 years from 10,400 households covering 361 clusters throughout Bangladesh. A multistage cluster-sampling procedure was adopted to conduct the survey. The survey obtained detailed information on fertility levels, marriage, fertility preferences, awareness, the use of family-planning methods, breastfeeding practices, nutritional status of women and young children, childhood mortality, maternal and child health, and knowledge and attitudes regarding HIV/AIDS and other sexually transmitted infections (STIs). The survey recorded 6,058 births occurred in the last five years preceding the survey. Such a large dataset provided a unique opportunity to study the use of facility-based delivery and socioeconomic factors affecting caesarean delivery. The details of the survey are given elsewhere (28).

### Variables

The dependent variables in this study are: ‘type of facility’ for childbirth and ‘type of delivery’, in particular, ‘caesarean section’ and ‘normal vaginal delivery (NVD)’. In the DHS questionnaire, the place of delivery was a variable with eleven different strata. In this study, the variable was made dichotomous by grouping into ‘type of facility’ (i.e. government hospital, *Upazilla* health complex, maternal and child welfare centre, private hospital or clinic, private medical centre, non-government static clinic, other non-government clinic and other public institutions with medical facilities) or ‘no facility’ (i.e., respondent's home, other's home, and others). The type of delivery was also made dichotomous as: ‘caesarean’ or ‘normal vaginal delivery’ (NVD). Independent variables included for analyses were: maternal age (15-19, >19-34, and >34-49 years), place of residence (urban and rural), women's education (no education, primary, secondary, and higher), religion (Islam and others), working status (not working and working), birth order (first, second, third, fourth, and fifth or above), year of birth (2002, 2003, 2004, 2005, 2006, and 2007), pregnancy intention status (wanted and unintended), births in the last five years preceding the survey (one and two or more), receiving skilled antenatal care (ANC) services, region, and wealth index.

### Statistical analyses

Data were made weighted by the corresponding weighting factor provided for each of the respondents in the survey. Binary logistic regression analyses were used for examining the effect of healthcare variables on the odds of facility-based delivery and caesarean birth. A woman who used any facility for childbirth was coded as ‘1’ and ‘0’ if otherwise. Besides, a woman whose delivery was performed by caesarean section was also coded as ‘1’ and ‘0’ if otherwise. Following the bivariate logistic regressions, multivariate logistic regression models were constructed separately for each of the dependent variables. The results of the regression analyses have been presented by odds ratios (ORs) with 95% confidence interval (CI). All statistical analyses in this study were performed by Statistical Package for Social Sciences (SPSS) version 17.

### Ethics

Since the BDHS data belong to the Ministry of Health and Family Welfare of the Bangladesh Government, a written permission was taken from all respondents in the study before starting the interview. Moreover, the BDHS followed all protocols prescribed by the World Health Organization (WHO). Hence, it was not necessary to take ethical approval from the Research Ethics Approval Committee of the Islamic University, Kushtia, Bangladesh.

## RESULTS

### Background characteristics of the respondents

Table 1 shows the percentage distribution of women by their sociodemographic characteristics. Of the women, one-third were adolescents, 61.5% were aged >19-34 years, and the rest were aged >34 years. Four of five women were rural residents. Over one-fourth had no formal education, 31.5% had primary education, 35.0% had secondary level of education, and only 6.0% had higher education. Slightly over one-fourth were working women. One-third of the births were of the first order, one-fourth were of the second order, and the rest were of third or higher order. Of the total births, 11.3% took place in 2002, 81.0% occurred during 2003-2006, and 7.7% occurred in 2007. One-third of the births were reported to be unintended (not wanted then and not wanted at all). A slightly over half of the women received ANC services. More women were from Dhaka division, followed by Rajshahi, Chittagong, Khulna, Sylhet and Barisal division. In terms of wealth index, 44.3% were poor, 19.4% were of middle class, and 36.4% were rich.

**T1able 1.** Sociodemographic characteristics of women, BDHS 2007

Characteristics	N=6,058	%
Maternal age (years)		
15-19	2,011	33.2
>19-34	3,728	61.5
>34-49	318	5.3
Residence		
Urban	1,249	20.6
Rural	3,809	79.4
Women's education		
No education	1,658	27.4
Primary	1,910	31.5
Secondary	2,122	35.0
Higher	366	6.0
Religion		
Islam	5,558	91.8
Other	499	8.2
Working status		
Not working	4,449	73.5
Working	1,606	26.5
Birth order		
First	2,050	33.8
Second	1,568	25.9
Third	1,009	16.7
Fourth	648	10.7
Fifth or higher	782	12.9
Year of birth		
2002	687	11.3
2003	1,197	19.8
2004	1,251	20.7
2005	1,265	20.9
2006	1,191	19.7
2007	466	7.7
Pregnancy intention status		
Unintended	4,027	66.5
Intended	2,025	33.5
Births in the last five years		
One	3,845	63.5
Two or more	2,212	36.5
Received skilled ANC		
No	2,365	48.3
Yes	2,535	51.7
Region		
Barisal	383	6.3
Chittagong	1,337	22.1
Dhaka	1,908	31.5
Khulna	578	9.5
Rajshahi	1,306	21.6
Sylhet	547	9.0
Wealth index		
Poorest	1,367	22.6
Poorer	1,312	21.7
Middle	1,173	19.4
Richer	1,149	19.0
Richest	1,056	17.4
Total	6,058	100.0

### Trends in institutional delivery and caesarean section

Table 2 shows the trends in ANC-seeking, institutional delivery, and caesarean sections for the period from 1993 to 2007. As shown in the table, the use of ANC services in Bangladesh has increased by two folds from 25.7% in 1993-1994 to 51.7% in 2007. By this time, the institutional delivery has increased more than four folds from 3.5% to 14.7%. The caesarean delivery has also increased by more than three folds from 2.4% in 1999-2000 to 7.5% in 2007.

### Prevalence and determinants of institutional delivery

The prevalence and association of institutional delivery with different socioeconomic characteristics are presented in Table 3. Overall, 14.7% of the babies were delivered at any medical facilities, with 7.6% in private hospitals or clinics, and 7.1% in government hospitals (not shown in the table). The bivariate logistic regression analyses applied on data showed strong significant associations between institutional delivery and place of residence, women's education, religion, working status of women, birth order, year of childbirth, pregnancy intention status, number of births in the last five years, receiving skilled ANC, region, and wealth index.

The women aged >19-34 years were more likely to report child delivery at any facility than women of other age-cohorts. Urban women, compared to their rural counterparts, were more likely to report the use of a facility for childbirth. Women's education showed a strong positive association with delivery at a medical facility. Birth order showed a negative association whereas year of childbirth showed a positive association with institutional delivery. Women with multiple pregnancies in the last five years preceding the survey were less likely to deliver at any medical facility. The women who sought ANC services were more likely to report undergoing institutional delivery than those who did not receive the ANC services. Institutional delivery was not uniform across the administrative regions of the country. The incidence of institutional delivery was higher among women of Khulna division, followed by Dhaka, Chittagong, Rajshahi, Barisal and Sylhet division. Wealth index showed a positive association with institutional delivery.

**T1able 2.** Trends in the use of any facility for delivery and caesarean section during 1993-2007

Year	Skilled ANC	Facility delivery	Caesarean section
	(%)	(%)	(%)
1993-1994	25.7	3.5	NA
1996-1997	26.4	4.1	NA
1999-2000	33.3	7.9	2.4
2004	48.8	9.3	3.5
2007	51.7	14.7	7.5

NA=Data not available

**T1able 3.** Results of logistic regression predicting likelihood of facility-based delivery, BDHS 2007

Characteristics	Home-delivery		Facility-based delivery		Unadjusted OR (95% CI)	p value	Adjusted OR (95% CI)	p value
	N	%	N	%				
Maternal age (years)^§^								
15-19	1,724	85.7	287	14.3	Reference	-	Reference	-
>19-34	3,158	84.8	566	15.2	1.08 (0.92-1.26)	0.346	0.98 (0.84-1.15)	0.831
>34-49	282	89.1	35	10.9	0.74 (0.51-1.07)	0.113	0.73 (0.49-1.07)	0.103
Residence^§^								
Urban	866	69.4	383	30.6	Reference	-	Reference	-
Rural	4,298	89.5	504	10.5	0.27 (0.23-0.31)	0.000	0.26 (0.22-0.31)	0.000
Women's education^‡^								
No education	1,610	97.3	44	2.7	Reference	-	Reference	-
Primary	1,767	92.6	141	7.4	2.92 (2.07-4.13)	0.000	1.50 (1.01-2.22)	0.042
Secondary	1,633	76.9	489	23.1	10.97 (8.00-10.05)	0.000	3.38 (2.32-4.92)	0.000
Higher	154	42.0	212	58.0	50.55 (35.11-72.77)	0.000	8.30 (5.34-12.99)	0.000
Religion^§^								
Islam	4,765	85.8	787	14.2	Reference	-	Reference	-
Other	400	80.1	99	19.9	1.51 (1.19-1.90)	0.001	1.59 (1.25-2.03)	0.000
Working status^§^								
Not working	3,732	84.0	713	16.0	Reference	-	Reference	-
Working	1,430	89.2	174	10.8	0.64 (0.53-0.76)	0.000	0.66 (0.55-0.79)	0.000
Birth order^‡^								
First	1,560	76.1	490	23.9	Reference	-	Reference	-
Second	1,335	85.2	233	14.8	0.56 (0.47-0.66)	0.000	0.66 (0.53-0.82)	0.000
Third	908	90.0	101	10.0	0.36 (0.28-0.45)	0.000	0.53 (0.40-0.70)	0.000
Fourth	609	94.3	37	5.7	0.19 (0.14-0.27)	0.000	0.48 (0.32-0.72)	0.000
Fifth or higher	752	96.6	26	3.4	0.11 (0.07-0.17)	0.000	0.44 (0.28-0.71)	0.001
Year of birth^§^								
2002	610	89.1	74	10.9	Reference	-	Reference	-
2003	1,075	89.9	121	10.1	0.92 (0.68-1.25)	0.608	0.91 (0.67-1.25)	0.575
2004	1,088	87.0	163	13.0	1.23 (0.92-1.64)	0.167	1.18 (0.87-1.59)	0.279
2005	1,063	84.0	203	16.0	1.57 (1.18-2.08)	0.002	1.52 (1.14-2.04)	0.005
2006	953	80.1	237	19.9	2.04 (1.54-2.70)	0.000	1.97 (1.48-2.63)	0.000
2007	376	80.8	89	19.2	1.95 (1.40-2.72)	0.000	1.76 (1.25-2.48)	0.001
Pregnancy intention status^†^							
Intended	3,383	84.0	643	16.0	Reference	-	Reference	-
Unintended	1,779	88.0	244	12.0	0.72 (0.62-0.84)	0.000	0.81 (0.69-0.95)	0.011
Births in the last five years^†^								
One	3,165	82.3	680	17.7	Reference	-	Reference	-
Two or more	1,762	90.6	182	9.4	0.48 (0.41-0.57)	0.000	0.52 (0.44-0.62)	0.000
Received skilled ANC^‡^							
No	2,285	96.6	80	3.4	Reference	-	Reference	-
Yes	1,827	72.1	708	27.9	11.03 (8.69-14.00)	0.000	4.41 (3.41-5.71)	0.000
Region^†^								
Barisal	346	90.5	36	9.5	Reference	-	Reference	-
Chittagong	1,155	86.4	182	13.6	1.51 (1.04-2.20)	0.032	1.60 (1.10-2.33)	0.015
Dhaka	1582	83.1	322	16.9	1.95 (1.36-2.80)	0.000	1.97 (1.37-2.84)	0.000
Khulna	448	77.6	130	22.4	2.77 (1.87-4.11)	0.000	2.61 (1.75-3.86)	0.000
Rajshahi	1,132	86.8	172	13.2	1.46 (1.00-2.13)	0.051	1.39 (0.95-2.03)	0.092
Sylhet	501	91.8	45	8.2	0.86 (0.54-1.36)	0.518	0.97 (0.61-1.54)	0.904
Wealth index^‡^								
Poorest	1,304	95.5	61	4.5	Reference	-	Reference	-
Poorer	1,239	94.7	69	5.3	1.19 (0.84-1.70)	0.330	0.76 (0.51-1.14)	0.182
Middle	1,069	91.1	104	8.9	2.09 (1.50-2.89)	0.000	0.98 (0.67-1.43)	0.916
Richer	953	83.0	196	17.0	4.41 (3.27-5.95)	0.000	1.49 (1.05-2.10)	0.026
Richest	598	56.6	458	43.4	16.43 (12.36-21.84)	0.000	3.84 (2.74-5.38)	0.000
Total	5,164	85.3	887	14.7	-	-	-	-

^§^Analysis adjusted for maternal age, residence, religion, working status, and year of childbirth;

^‡^Analysis adjusted for women's education, birth order, antenatal care-seeking, and wealth index;

^†^Analysis adjusted for pregnancy intention status, number of childbirths in the last five years, and region

To ease the interpretation, adjusted ORs with 95% CI have been expressed as model-based predicted probabilities of institutional delivery. The multivariate analysis revealed that, after being adjusted for other socioeconomic factors, maternal age, place of residence, religion, working status of women, birth order, year of childbirth, pregnancy intention status, number of pregnancy in the last five years preceding the survey, receiving skilled ANC services, region, and wealth index had significant effects on institutional delivery. The adjusted ORs as shown in Table 3 suggest that women's education was the most important significant factor in analyzing the institutional delivery. The higher was the level of education, the higher was the use of institutional delivery. The adolescents, compared to their older counterparts, were more likely to use medical facilities for childbirth. The rural women, compared to their urban counterparts, were less likely to deliver at any medical facilities.

The preference for institutional delivery was higher among the non-Muslim women than their Muslim sisters. Compared to working women, the non-working women were more likely to deliver at medical institutions. Birth order was negatively associated with the likelihood of institutional delivery. The calendar year of childbirth revealed that, after controlling for maternal age, place of residence, religion, working status of women, the likelihood of institutional delivery has increased in the recent years. Higher-order births among women within the last five years preceding the survey showed a significantly low rate of institutional delivery than women who delivered only once. ANC-seeking had a strong positive effect on institutional delivery. The region showed heterogeneous likelihood of institutional delivery among women. As expected, wealth index had significantly a net positive effect on the use of medical facility for childbirth.

### Prevalence and determinants of caesarean section

Table 4 shows the prevalence of caesarean section, along with the results of bivariate and multivariate logistic regression analyses. Overall, 7.9% births were through caesarean sections during 2002-2007. The bivariate analyses applied in the study showed that maternal age, place of residence, women's education, religion, working status of women, birth order, year of childbirth, pregnancy intention status, number of childbirths in the last five years preceding the survey, ANC-seeking, region, and wealth index were significantly associated with caesarean section.

**T1able 4.** Logistic regression results predicting likelihood of caesarean section, BDHS 2007

Characteristics	Vaginal delivery		Caesarean delivery		Unadjusted OR (95% CI)	p value	Adjusted OR (95% CI)	p value
	N	%	N	%				
Maternal age (years)^§^								
15-19	1,881	93.5	130	6.5	Reference	-	Reference	-
>19-34	3,398	91.2	328	8.8	1.40 (1.13-1.72)	0.002	1.30 (1.05-1.61)	0.016
>34-49	298	93.4	21	6.6	1.02 (0.63-1.64)	0.945	1.03 (0.63-1.67)	0.921
Residence^§^								
Urban	1,046	83.8	202	16.2	Reference	-	Reference	-
Rural	4,531	94.2	277	5.8	0.32 (0.26-0.38)	0.000	0.32 (0.26-0.39)	0.000
Women's education^‡^								
No education	1,632	98.4	27	1.6	Reference	-	Reference	-
Primary	1,855	97.2	53	2.8	1.74 (1.09-2.79)	0.021	1.11 (0.63-1.94)	0.730
Secondary	1857	87.5	265	12.5	8.76 (5.85-13.11)	0.000	2.77 (1.64-4.67)	0.000
Higher	232	63.4	134	36.6	35.38 (22.83-54.84)	0.000	5.79 (3.27-10.25)	0.000
Religion^§^								
Islam	5,136	92.4	420	7.6	Reference	-	Reference	-
Other	441	88.4	58	11.6	1.61 (1.20-2.15)	0.001	1.64 (1.22-2.21)	0.001
Working status^§^								
Not working	4,062	91.3	386	8.7	Reference	-	Reference	-
Working	1513	94.3	92	5.7	0.64 (0.51-0.81)	0.000	0.66 (0.52-0.83)	0.001
Birth order^‡^								
First	1,783	87.0	267	13.0	Reference	-	Reference	-
Second	1,441	91.9	127	8.1	0.59 (0.47-0.73)	0.000	0.70 (0.54-0.91)	0.008
Third	958	95.2	48	4.8	0.34 (0.25-0.46)	0.000	0.46 (0.31-0.66)	0.000
Fourth	624	96.3	24	3.7	0.26 (0.17-0.39)	0.000	0.63 (0.38-1.03)	0.066
Fifth or higher	771	98.5	12	1.5	0.10 (0.06-0.19)	0.000	0.36 (0.17-0.74)	0.006
Year of birth^§^								
2002	644	93.9	42	6.1	Reference	-	Reference	-
2003	1,128	94.2	69	5.8	0.94 (0.64-1.40)	0.778	0.95 (0.63-1.41)	0.782
2004	1,164	93.0	87	7.0	1.16 (0.79-1.70)	0.445	1.10 (0.75-1.62)	0.620
2005	1,160	91.8	104	8.2	1.38 (0.95-2.00)	0.089	1.33 (0.91-1.93)	0.142
2006	1,066	89.5	125	10.5	1.81 (1.26-2.60)	0.001	1.71 (1.18-2.47)	0.005
2007	414	89.0	51	11.0	1.92 (1.25-2.94)	0.003	1.69 (1.10-2.62)	0.017
Pregnancy intention status^†^							
Intended	3,685	91.6	339	8.4	Reference	-	Reference	-
Unintended	1,886	93.1	139	6.9	0.80 (0.65-0.98)	0.034	0.89 (0.73-1.10)	0.293
Births in the last five years^†^							
One	3,474	90.4	369	9.6	Reference	-	Reference	-
Two or more	2,103	95.0	110	5.0	0.49 (0.39-0.61)	0.000	0.51 (0.41-0.65)	0.000
Received skilled ANC^‡^							
No	2,335	98.7	30	1.3	Reference	-	Reference	-
Yes	2,141	84.5	391	15.5	14.12 (9.71-20.54)	0.000	4.96 (3.33-7.39)	0.000
Region^†^								
Barisal	368	96.1	15	3.9	Reference	-	Reference	-
Chittagong	1,245	93.1	92	6.9	1.80 (1.03-3.14)	0.039	1.90 (1.09-3.33)	0.024
Dhaka	1,705	89.4	202	10.6	2.89 (1.69-4.94)	0.000	2.92 (1.71-5.00)	0.000
Khulna	521	90.3	56	9.7	2.61 (1.46-4.69)	0.001	2.46 (1.37-4.43)	0.003
Rajshahi	1,218	93.3	88	6.7	1.77 (1.01-3.09)	0.046	1.67 (0.96-2.96)	0.067
Sylhet	520	95.2	26	4.8	1.24 (0.65-2.37)	0.511	1.41 (0.74-2.70)	0.299
Wealth index^‡^								
Poorest	1,337	97.8	30	2.2	Reference	-	Reference	-
Poorer	1,289	98.2	24	1.8	0.81 (0.47-1.39)	0.330	0.70 (0.38-1.28)	0.249
Middle	1,127	96.1	46	3.9	1.78 (1.12-2.84)	0.000	0.90 (0.52-1.57)	0.722
Richer	1,046	91.1	102	8.9	4.31 (2.85-6.52)	0.000	1.67 (1.01-2.73)	0.044
Richest	778	73.8	277	26.2	15.67 (10.66-23.03)	0.000	4.03 (2.49-6.51)	0.000
Total	5,577	92.1	478	7.9	-	-	-	-

^§^Analysis adjusted for maternal age, residence, religion, working status, and year of childbirth;

^‡^Analysis adjusted for women's education, birth order, antenatal care-seeking, and wealth index;

^†^Analysis adjusted for pregnancy intention status, number of childbirths in the last five years, and region

The results of the multivariate logistic regression analysis revealed that, when other socioeconomic factors were held constant, maternal age, place of residence, religion, working status, birth order, year of childbirth, pregnancy intention status, and higher birth order in the last five years, receiving ANC services, region, and wealth index were found to have significant effect on caesarean delivery. The only variable ‘pregnancy intention status’ had no significant effect on caesarean delivery after controlling for higher birth order in the last five years and place of region. Findings revealed that the likelihood of the use of caesarean section was significantly attenuated when other independent variables were held constant.

The women aged >19-34 years were more likely to undergo caesarean delivery than others. The rural women, compared to their urban counterparts, were less likely to deliver through caesarean sections. The likelihood of caesarean delivery increased with increase in the level of women's education. Birth order showed a consistent decrease in the likelihood of caesarean section compared to NVD. The non-Muslim and non-working women were more likely to undergo caesarean delivery. The likelihood of caesarean delivery increased significantly during 2006-2007 compared to 2002. The women who had experienced more than one pregnancy during the last five years were less likely to undergo caesarean section. The women who sought skilled ANC services were more likely to use caesarean sections for childbirth. The women from Chittagong, Dhaka, Khulna and Rajshahi division were significantly more likely to use caesarean sections for delivery than women of Barisal division. The non-poor women, compared to the poor, were more likely to undergo caesarean sections for childbirth.

## DISCUSSION

This study aimed to explore the socioeconomic factors affecting the preference for child-delivery settings and caesarean sections in Bangladesh. Findings showed that, although half of the women used ANC services, only 14.7% births took place in medical facilities, and 7.9% deliveries were performed by caesarean sections. The findings revealed inequities in safe delivery practices by socioeconomic strata in Bangladesh. Women with higher economic status and higher level of education used more opportunities of the appropriate place for delivery and underwent caesarean sections. The proportion of institutional delivery and caesarean sections increased with the increase in women's education and wealth. Besides, ANC-seeking was also a vital predictor of institutional delivery and caesarean sections. Among other variables included in the analyses, place of residence, religion, working status of women, birth order, pregnancy intention status, higher birth order in the last five years, and regions appeared as important determinants of institutional delivery.

Consistent with earlier studies (29), our findings showed that women aged >19-34 years, compared to those of 15-19 years, were more likely to undergo caesarean section whereas the adolescents were more likely to use medical facilities for childbirth than women of other age-cohorts. The findings of this study revealed that women's education was the strongest predictor of institutional delivery and caesarean sections for childbirth in Bangladesh. Similar findings have been reported from small-scale surveys in Bangladesh (30) and other developing countries (2,31). A study on rural Bangladeshi women reported that only a small proportion of deliveries took place in the hospitals or clinics, and this proportion was higher among women with secondary or higher education and those who received ANC services (32). These findings are attributed to the significant changes in women's education in the recent years. Higher-educated women are more informed of the costs and benefit of the use of maternal healthcare services (33). Education is likely to enhance female autonomy such that women develop greater confidence and capability to make decisions about their own health (33,34). It is also likely that educated women seek higher-quality services and have greater ability to use healthcare inputs that offer better care (35).

Our study showed that urban women were significantly more likely to use medical settings and caesarean sections. Irrespective of the place of residence, women and their families generally do not prefer caesarean delivery unless women feel serious obstetric complications. Besides, preference for delivery at medical settings and caesarean sections among urban residents may be attributed to easy accessibility, availability of government, private and non-government medical facilities for maternal and child healthcare. Obstetric surgery is a necessary component of the safe motherhood services but it can also lead to exploitation of women and families due to the emergency nature of some complications requiring expensive surgery (36). Lifesaving caesarean section is a basic need of any woman with complicated pregnancy. This should not be influenced by any social or economic factor. It should also be considered whether caesarean section is ‘lifesaving’ for the mother and/or the newborn or is ‘unnecessary and risk-producing’. However, our findings are consistent with many earlier studies conducted elsewhere (36,37,38,39).

Consistent with earlier studies (40), the observation that Muslim mothers sought lesser assistance from medical settings than non-Muslim mothers is likely to be attributed to the religious beliefs, cultural norms, and traditional practices. Muslim husbands do not permit their wives to go to doctors or outside their home for the breach of privacy. The element of choice becomes more important in Bangladeshi sociocultural ambiance where women are unwilling to be examined by a male physician and sometimes by unfamiliar nurses in healthcare facilities. Moreover, the health facilities in Bangladesh still have lack of sufficient female health personnel, and there is an increasing demand that medical care for women should be provided by women. Surprisingly, working women were less likely to avail of the opportunity of the use of institutional delivery and caesarean section than their non-working counterparts. Perhaps working women experience time constraints that reduce their opportunities for receiving antenatal care (41). However, this would not explain why they are less likely than other women to receive skilled delivery care. This finding urges further investigation into what mechanisms influence working women compared to the non-working women for lesser use of medical facilities as well as caesarean sections.

In the recent years, there has been an increasing tendency among pregnant women without obstetric indications for caesarean section because they perceive it to be safe and more convenient than NVD (42). This situation has become a significant factor, leading to the increased rate of skilled maternity care services, including institutional delivery and caesarean section in developing countries (19). This has been reflected from our findings that both institutional delivery and caesarean sections have increased in the recent years. The increasing trend over time in the use of medical facilities and caesarean sections for childbirth may be partly attributed to economic transition, modernization process, and women's increased level of education in Bangladesh.

Findings of our study revealed that women with first-order pregnancy were more likely to have institutional delivery and caesarean sections than those with second or higher-order birth and women with single birth in the last five-years. This reflects women's perceptions regarding the efficacy of the procedure as a means to ensure newborn survival and to avert the risks of birth complications or stillbirth. Consistent with our findings, studies showed that women are increasingly inclined to opt for delivery by caesarean section for non-medical reasons, such as fear of labour pain, concerns about date or time of birth that are traditionally believed to be auspicious, and the belief that delivery by caesarean section ensures protection of the baby's brain (43). An apparent increase in the incidence of uterine ruptures and concern about maternal and foetal safety has challenged the choice of vaginal delivery by women having a scarred uterus. As a consequence, clinicians are increasingly being faced with difficulty in deciding the mode of delivery for women whose first delivery was performed by caesarean section (44). Whether the caesarean section results in the best outcome for mothers and children continues to be a matter of debate. It is evident that a better outcome (including lower morbidity and mortality) does not necessarily result from caesarean sections (19).

A possible factor identified to have significant effect on maternity care is the pregnancy intention status. This finding is also consistent with earlier studies (40). Women with unintended pregnancies may initially attempt to end their pregnancies and conceal from others (40). As a result, women with unintended pregnancy become less motivated to seek ANC compared to their counterparts who reported their pregnancy as wanted. Higher number of pregnancy during the last five years is also another important factor that has been identified to have negative effect on institutional delivery and caesarean sections. Multiparities may affect the use of healthcare in several ways. For example, having had the experience of previous pregnancy may provide a woman with the idea that she already knows what may happen during the prenatal period and at the time of delivery and, hence, do not need to see a care provider or undergo caesarean section. Another possible reason may be that women with high parity have developed confidence and may believe that healthcare is not that necessary due to the experience and knowledge accumulated from previous pregnancies and births (40). This finding is also consistent with earlier studies (40,45).

The use of ANC services was a strong predictor for both institutional delivery and caesarean section. The associations between ANC and caesarean delivery may contain some elements of self-selection. The women who received ANC come in contact with healthcare providers who are likely to encourage them to give birth in a medical facility. For example, women with high-risk pregnancies and identified problems are presumably more likely to be advised to have more consultations and investigations, such as ultrasound (46). On the other hand, it is likely that increased use of ANC services leads women to increase care-seeking, including a greater openness to caesarean delivery. This finding has important programmatic implications. This suggests that it is possible to promote institutional delivery by expanding antenatal care coverage and associated counselling. Indeed, many of the factors influencing maternal behaviours, such as fear of pain, are meaningful precisely because these are understood to differ by socioeconomic status and to be embedded in discriminating practices (47).

The analyses of this study suggest that the use of maternal healthcare services, in particular institutional delivery and childbirth through caesarean section, is not uniform across the administrative regions of the country. The women of Chittagong, Dhaka, Khulna and Rajshahi division were significantly more likely to avail of institutional delivery and caesarean sections than women of Barisal division whereas Sylhet division did not show significant difference in the likelihood of the use of these two maternal healthcare services. This may be partly attributed to heterogeneous socioeconomic status and women's education in the regions. In addition, one of the major causes of regional variations in the use of maternity care services is the imbalance of health workforce across the regions. It is evident that the Barisal, Chittagong and Sylhet division have higher rates of vacancies of service providers, and the use of maternity care services in these particular divisions is lower (33). Besides, Dhaka, Khulna and Rajshahi division have less vacant posts for healthcare providers, resulting in the higher use of maternity care services in these administrative regions (33).

Aside from the medical benefits and risks of caesarean delivery for individual women, an important concern is the economic factor. The lower use of institutional and caesarean delivery is often attributed to the relatively higher costs, which has reflected from the economic status of the women measured by wealth index. For instance, the higher was the wealth index, the higher was the use of skilled services for childbirth. Thus, one of the main factors that influence the use of medical facilities and caesarean sections is possibly financial exploitation. This phenomenon is described in other countries as ‘reverse equity’ where women with higher wealth index, presumably with less medical risks, have higher caesarean rates (48). In contrast, a study showed to have no effect of economic status, defined by size of landholdings across the three groups in Bangladesh (49). Instead, factors that affected these practices included the education of the woman and of her husband, complications of delivery, and receipt of ANC (49). However, our findings are in good agreement with those of earlier studies conducted elsewhere (37,38,50).

### Limitations

The study has several limitations. For example, besides the selected demographic, socioeconomic and cultural factors which have been included in this analysis, a host of other programmatic factors, e.g. accessibility, quality, and costs of delivery services, and cultural factors, e.g. religiosity, prejudices, women's role in decision-making process, and subordinate status of women are also likely to influence the delivery practices of women. However, due to lack of relevant data, the effects of these programmatic and cultural factors on the child-delivery practices could not be examined in this study. In addition, the available data did not permit us to examine all aspects of the delivery practices. Despite these limitations, the strength of the study is that it dealt with a nationally-representative data with large sample-size.

### Conclusions

The rates of utilization of institutional delivery and caesarean sections are very low in Bangladesh. However, fewer caesarean sections do not necessarily mean lower healthcare quality (51). The appropriate range for the caesarean section rate in a country remains a matter of debate. However, the recommended lower limit ranges from a minimum of 1% to an optimum target of 5% to avoid death and severe morbidity in the mother. Although these figures are good estimates based on complication rates in the mother and on historical data, it is unknown whether the frequency of intervention is enough to prevent avoidable perinatal deaths (51). The best known recommended upper limit is 15% suggested by WHO. The study identified several important factors that significantly affect the use of medical facilities and caesarean section for childbirth; these are women's education, birth order, ANC-seeking, region, and wealth index. Besides formal education, women should be informed of the long-run benefits of the use of maternal healthcare and demerits of traditional birth practices through informal education. Health education programmes on the risks of caesarean delivery and benefits of NVD should be conducted among women and society at large. Women should be encouraged and advised for regular ANC check-up for the overall well-being of both mother and child. Special programmes should be undertaken to have easy accessibility for maternal healthcare-seeking across the regions of the country. Because higher proportions of institutional deliveries take place in the private-sector than in the public-sector facilities, efforts should also be made to strengthen private-sector health facilities to make them more accessible to rural mothers in terms of availability and quality of services and costs. Finally, there is a need for further investigation to examine the effects of some programmatic factors (e.g. accessibility, quality, and cost of delivery services) and cultural factors (e.g. religiosity, prejudices, women's role in the decision-making process, subordinate status of women) on the safe delivery practices of women in Bangladesh.
